# Nrf2 orchestrates transition from acute to chronic otitis media through inflammatory macrophages

**DOI:** 10.3389/fimmu.2023.1170388

**Published:** 2023-04-14

**Authors:** Wenyan Fan, Hongming Xu, Chenling Shen, Jia Fang, Xiaoyan Li

**Affiliations:** Department of Otolaryngology, Shanghai Children’s Hospital, School of Medicine, Shanghai Jiao Tong University, Shanghai, China

**Keywords:** acute otitis media (AOM), chronic otitis media (COM), macrophages, macrophage polarization, nuclear factor erythroid 2-related factor 2 (Nrf2) signaling pathway

## Abstract

**Introduction:**

Acute and chronic otitis media (AOM and COM) are common middle ear infections that can lead to hearing loss and other complications. Recent research has shown that both macrophages and nuclear factor erythroid 2-related factor 2 (Nrf2) signaling pathway are involved in the immune response to and the resolution of otitis media. However, the specific effects of Nrf2 on macrophages in the transition of AOM to COM are not well understood, and a practical approach to prevent this transition by targeting Nrf2/macrophages has not been established.

**Methods:**

In an AOM mouse model using lipopolysaccharide (LPS) injection into the middle ear, middle ear effusion (OME)-macrophages were isolated and analyzed for Nrf2 expression. M2-like polarization of macrophages was induced by Nrf2 activation and its effects on inflammatory resolution were studied by examining inflammatory neutrophils and macrophages, proinflammatory cytokines, and oxidative levels. The survival of human middle ear epithelial cells (HMMECs) co-cultured with Nrf2-modified macrophages was also evaluated. Furthermore, restoration of Nrf2 in macrophages with adeno-associated virus (AAV) vectors was performed to determine the effect on the transition of AOM to COM in experimental mice.

**Results:**

Reduced Nrf2 in OME-macrophages during the recovery phase was associated with uncured AOM or its development into COM, demonstrated by persistent increases in inflammatory neutrophils and macrophages, proinflammatory cytokines, and oxidative levels. Nrf2 activation induced M2-like polarization of macrophages, which improved the survival of co-cultured HMMECs treated with LPS *in vitro*. Restoration of Nrf2 in OME-derived low-Nrf2-expressing macrophages with AAV vectors significantly inhibited the transition of AOM to COM in experimental mice.

**Discussion:**

Nrf2 in macrophages plays a critical role in the immune response to and resolution of otitis media Restoration of Nrf2 expression in OME-macrophages could be a promising therapeutic approach to prevent the development of COM in AOM patients.

## Introduction

Acute and chronic otitis media (AOM and COM) are common ear infections, especially in children (1). AOM commonly occurs in children under the age of 5, and is characterized by the rapid onset of symptoms, including ear pain, fever, and hearing loss (2). COM is defined as an ear infection that persists for more than 6 weeks, and can lead to more severe complications, including hearing loss, vertigo, and even facial paralysis (2). COM is more common in adults, particularly in those with underlying conditions that affect the immune system, such as diabetes (2). The major pathological changes in otitis media include inflammation, fluid buildup, and damage to the middle ear structures (3).

Macrophages are a type of inflammatory cells that participate in both innate and adaptive immune responses to infection (4). Depending on the signals they receive from the surrounding tissue microenvironment, macrophages can adopt different activation states or phenotypes, which are generally classified as proinflammatory M1 and anti-inflammatory M2 macrophages (4). M1 macrophages are classically activated by pro-inflammatory cytokines and microbial products such as lipopolysaccharide (LPS). They produce pro-inflammatory cytokines and chemokines, and have enhanced microbicidal activity, making them important for the clearance of infections. M2 macrophages are alternatively activated by anti-inflammatory cytokines, growth factors, and apoptotic cells (5). They produce anti-inflammatory growth factors and cytokines for a proper tissue repair and remodeling. In AOM, macrophages are typically activated to an M1 phenotype, which is characterized by the production of proinflammatory chemokines and cytokines for recruiting other immune cells to the site of infection (6). M1 macrophages also have enhanced microbicidal activity, which helps to clear the infection. In COM, however, macrophages may shift to an M2 phenotype to produce anti-inflammatory growth factors and cytokines to promote tissue healing. On the other hand, M2 macrophages may also contribute to persistent inflammation and tissue damage if the infection is not cleared (6). In addition, M2 macrophages are less effective at killing bacteria and other pathogens, which can further contribute to disease progression (7). However, distinction between M1 and M2 macrophages is rather artificial. In fact, macrophages can exhibit a range of activation states in response to the inflammatory tissue microenvironment (7). The timely differentiation and polarization of macrophages in AOM may be critical for disease control and prevention of its progression to COM (7).

Nuclear factor erythroid 2-related factor 2 (Nrf2) is a critical mediator of cellular defense against inflammation and oxidative stress (8). In response to oxidative stress, activation and subsequent nuclear translocation of Nrf2 occur, followed by its binding to antioxidant response element (ARE) sequences in the target gene promoter to initiate transcription of antioxidant enzymes, detoxification enzymes, and other proteins involved in cellular defense mechanisms (9). The NRF2 signaling pathway has been shown to regulate inflammation in AOM and COM (10). However, it is not known whether this regulation is mainly through macrophages. Moreover, a practical approach to prevent the transition from AOM to COM by targeting Nrf2/macrophages has not been established. These questions were addressed in the current study (11).

## Materials and methods

### Ethics

The animal experiments were conducted following the guidelines for laboratory animal care and use approved by Shanghai Children’s Hospital. Male C57BL/6 mice, aged 10 weeks and weighing between 18-22g, were procured from the Shanghai Laboratory Animal Research Center in China.

### Cell culture and co-culture

Human middle ear epithelial cells (HMMECs) were purchased from ScienCell (#2460) and cultured in collagen-coated culture flasks in a humidified incubator at 37°C with 5% CO_2_. The media was 1:1 mixture of Dulbecco’s modified Eagle’s medium (DMEM) and Ham’s F12 medium, supplemented with transferrin, insulin, hydrocortisone, epidermal growth factor (EGF) and cholera toxin. To ensure optimal growth and viability, HMMECs were passaged before reaching confluency and sub-cultured every 3-4 days using trypsin. 3T3 cell line was cultured in DMEM with 5% FBS. The extraction of mouse bone marrow-derived macrophages (BMDMs) involved utilizing a 25-gauge needle and DMEM medium to flush the bone marrow out of the bone. The bone marrow cells were collected in a sterile tube to be centrifuged at 300g for 5 minutes to pellet the cells. The cells were then resuspended in DMEM medium containing 10% FBS and 1% penicillin-streptomycin to be cultured at 37°C and 5% CO2 for 3 days, with the addition of L929 cell (American Type Culture Collection, ATCC, Rockville, MD, USA)-conditioned media to DMEM by 1:10 on the first day to stimulate differentiation into macrophages. L929 cell-conditioned media are commonly used to induce macrophage differentiation in bone marrow-derived cells. L929 cells produce macrophage colony-stimulating factor (M-CSF), which stimulates the differentiation of bone marrow cells into macrophages. To prepare L929-conditioned media for macrophage differentiation, culture L929 cells until 80-90% confluency, then incubate them in fresh complete growth medium for 7-10 days. Collect, filter, and store the L929-conditioned medium containing M-CSF. Finally, culture bone marrow-derived cells in a mixture of L929-conditioned medium and fresh complete growth medium to induce macrophage differentiation over 5-7 days, refreshing the medium every 2-3 days. On the third day, BMDMs were harvested. An CCK-8 assay (Millipore) was applied to assess viable cells. Co-culture was performed in a transwell.

### Generation of macrophage-specific Nrf2-correcting vectors

A complete coding sequence for mouse Nrf2 cloned from total cDNA of mouse BMDMs and a backbone plasmid containing a CD11b promoter purchased from Addgene (#26168; 5′ cloning site: HindIII, 3′ cloning site: SmaI) (12) were used for generating macrophage-specific Nrf2-correcting vectors: overexpressing construct, pCD11b-Nrf2; control construct, pCD11b-scramble. These constructs were used to generate an adeno-associated virus (AAV, serotype 6) carrying Nrf2 under a macrophage-specific CD11b promoter (AAV-pCD11b-Nrf2) and a control AAV carrying a scramble sequence (Scr) under the CD11b promoter (AAV-pCD11b-Scr) with transfection using Lipofectamine 3000 reagent (Invitrogen). Both viruses also carried a green fluorescent protein (GFP) as a reporter connected with Nrf2 or Scr with a p2A construct to allow expression of both Nrf2/Scr and GFP controlled by the CD11b promoter.

### Animal work

The mice were housed in a controlled environment at a constant temperature of 20 ± 3°C and relative humidity of 45%, with 12-hour light/dark cycles. To induce AOM, the mice received an injection of LPS (1.0 mg/ml, Sigma, USA) into the middle ear of the right ear. For assessing the LPS-induced AOM, mice were divided into 2 groups. In control group, mice received saline injection. In experimental group, mice received LPS. For assessing the effects of AAV-pCD11b-Nrf2 on macrophages and AOM solution, mice were selected from those had low-Nrf2-expressing (otitis media with effusion, a collection of fluid in the middle ear space) OME-macrophages at day7. These mice were evenly divided into 2 groups, with one group of the mice receiving LPS injection at day 0 and AAV-pCD11b-Scr injection at day 7, and another group of the mice receiving LPS injection at day 0 and AAV-pCD11b-Nrf2 injection at day 7. OME extraction and viral injection (10^10^ in 10µl) were both assisted by myringotomy. Blood serum samples were obtained from mouse tail vein. At sacrifice, mouse middle ear tissue was obtained.

### ROS measurement

A DCFH-DA assay (2044-85-1, Sigma-Aldrich) was used to measure reactive oxygen species (ROS) levels. In short, this test used a dye called DCFH-DA that can enter cells and turn into a fluorescent compound (DCF) when interacting with ROS. The relative intensity of DCF fluorescence in DCFH-DA-treated cells was measured by fluorescence spectroscopy to represent the ROS levels in the samples.

### MDA measurement

The thiobarbituric acid reactive substances (TBARS) assay (ab118970, Abcam) was used to measure levels of Malondialdehyde (MDA), a marker of lipid peroxidation that is produced when free radicals attack and degrade cell membrane lipids. Briefly, the sample was homogenized in a buffer to release the lipid components. To a known volume of sample, a solution of trichloroacetic acid (TCA) and thiobarbituric acid (TBA) was added to react with MDA to form a colored compound. After heating, the sample was cooled and centrifuged to remove any precipitate that has formed. The intensity of the colored compound was measured using a spectrophotometer. The absorbance is proportional to the MDA concentration in the sample.

### SOD measurement

Superoxide dismutase (SOD) is an enzyme to converse superoxide anion to hydrogen peroxide, which can then be further broken down by other enzymes or antioxidants. A nitroblue tetrazolium (NBT) assay (N6495, ThermoFisher Scientific) was used to measure SOD levels. The sample was homogenized to release the enzyme, afterwards a reaction mixture containing xanthine oxidase (XO), which generates superoxide anions that react with NBT to produce a blue formazan product, was added. The sample was incubated at 37°C for 20 minutes and then stopped by adding ethanol that solubilized the formazan product and stopped the enzymatic reaction. The intensity of the blue color was measured using a spectrophotometer. The absorbance is proportional to the amount of superoxide anions produced in the reaction, which is inversely proportional to the SOD activity in the sample.

### GSH measurement

Glutathione (GSH) is a tripeptide antioxidant that scavenges ROS to affect redox reactions. The GSH levels was measured based on production of a yellow compound in the reaction of GSH with Ellman’s reagent (5,5’-dithiobis-(2-nitrobenzoic acid); DTNB). Briefly, the homogenized sample was added with Ellman’s reagent, which reacts with GSH to produce a yellow compound called 5-thio-2-nitrobenzoic acid (TNB). The sample was incubated for 10 minutes at room temperature, and then stopped by adding trichloroacetic acid that stabilized the TNB product and prevented further reaction. The intensity of the yellow color was measured using a spectrophotometer. The absorbance is proportional to the GSH concentration in the sample.

### Arginase activity

Arginase catalyzes the conversion of arginine to ornithine and urea. In order to measure arginase activity, 10-20 µL of lysate or homogenate was mixed with 50 mM Tris-HCl buffer (pH 7.5) containing 10 mM MnCl2 and incubate at 37°C for 10 minutes. Afterwards, 0.5 M HCl was added to stop the reaction and transfer the reaction mixture to a clean microcentrifuge tube. Next, 25% (w/v) trichloroacetic acid was added to the reaction mixture and centrifuge at 13,000 rpm for 10 minutes at 4°C to remove any protein precipitates. The supernatant was then mixed with an equal volume of 0.5 M α-isonitrosopropiophenone in ethanol and incubate at 90°C for 45 minutes to form a colored product. Absorbance at 540 nm was measured using a spectrophotometer. Finally, the amount of produced urea was measured using a standard urea solution and normalized to the protein concentration of the lysate or homogenate.

### Counting neutrophils using a hemocytometer

The cell suspension was collected, mixed thoroughly and then transferred a small volume of the sample to a clean 1.5 ml microcentrifuge tube. Dilute the sample with PBS to avoid overcrowding of cells on the hemocytometer. Add 0.4% trypan blue to the diluted cell suspension to stain dead cells. Load the hemocytometer with the diluted cell suspension using a pipette, being careful to avoid air bubbles. Place the loaded hemocytometer under the microscope and focus the lens on the grid lines. Starting from one corner of the hemocytometer, count the number of cells within the grid lines under 40x magnification. Repeat the counting process for each of the 4 corner squares of the hemocytometer. Calculate the total number of cells in the 4 squares and multiply by the dilution factor to obtain the total number of cells in the original sample. For Counting neutrophils were visualized with a segmented nucleus and cytoplasmic granules. Record the number of neutrophils counted. Divide the number of neutrophils counted by the total number of cells and multiply by 100 to obtain the percentage of neutrophils in the sample.

### Flow cytometry

Mouse middle ear tissue was digested with 0.25% trypsin (Invitrogen) and 5mg/ml DNase (Invitrogen) for 20 minutes to generate a single cell fraction. Macrophages were isolated from this single cell preparation by fluorescence activated cell sorting (FACS) based on F4/80 positivity. CD163 was used to distinguish M1 (negative) from M2 (positive) macrophages. All fluorescence-conjugated antibodies were purchased from Becton-Dickinson Biosciences. Apoptosis was assessed using an Annexin V-apoptosis analysis kit (Invitrogen). The flow cytometry data were analyzed and presented by Flowjo (Flowjo LLC, Ashland, OR, USA).

### Quantitative real-time PCR (RT-qPCR)

To prepare cDNA for RT-qPCR, RNA was extracted using an RNeasy kit (Qiagen, Beijing, China) with Qiagen pre-designed primers. The expression values for the examined genes were normalized using β-actin as a housekeeping gene, and quantification was performed using a 2-△△Ct method.

### ELISA and immunostaining

Total protein was extracted to be used in ELISA assays for mouse Nrf2 (MBS7612500, MyBioSource), IL-1β (ab197742; Abcam), iNOS (MAB9502, R&D Systems), tumor necrosis factor alpha (TNFα, ab208348; Abcam), IL-6 (ab222503, Abcam), arginase 1 (ARG1, ab269541; Abcam), interferon gamma (IFNɣ, ab282874; Abcam), and CD163 (ab272204; Abcam). Immunocytochemistry for cleaved caspase 3 and caspase 9 was done using rabbit anti-mouse antibodies (Abcam).

### Statistical analysis

Analysis of multiple groups was performed using one-way ANOVA. If a significant difference was detected, a *post-hoc* Bonferroni test was used to identify the specific pairs of groups with significant differences between their means. Correlation analysis was performed using Pearson correlation coefficient analysis. Individual values were presented in the Graphs generated by GraphPad Software, version 7 (Inc. La Jolla, CA, USA). The statistical significance level was set at p<0.05, which was indicated by an asterisk (*).

## Results

### LPS injection into middle ear induces AOM in mice

**Figure 1:**
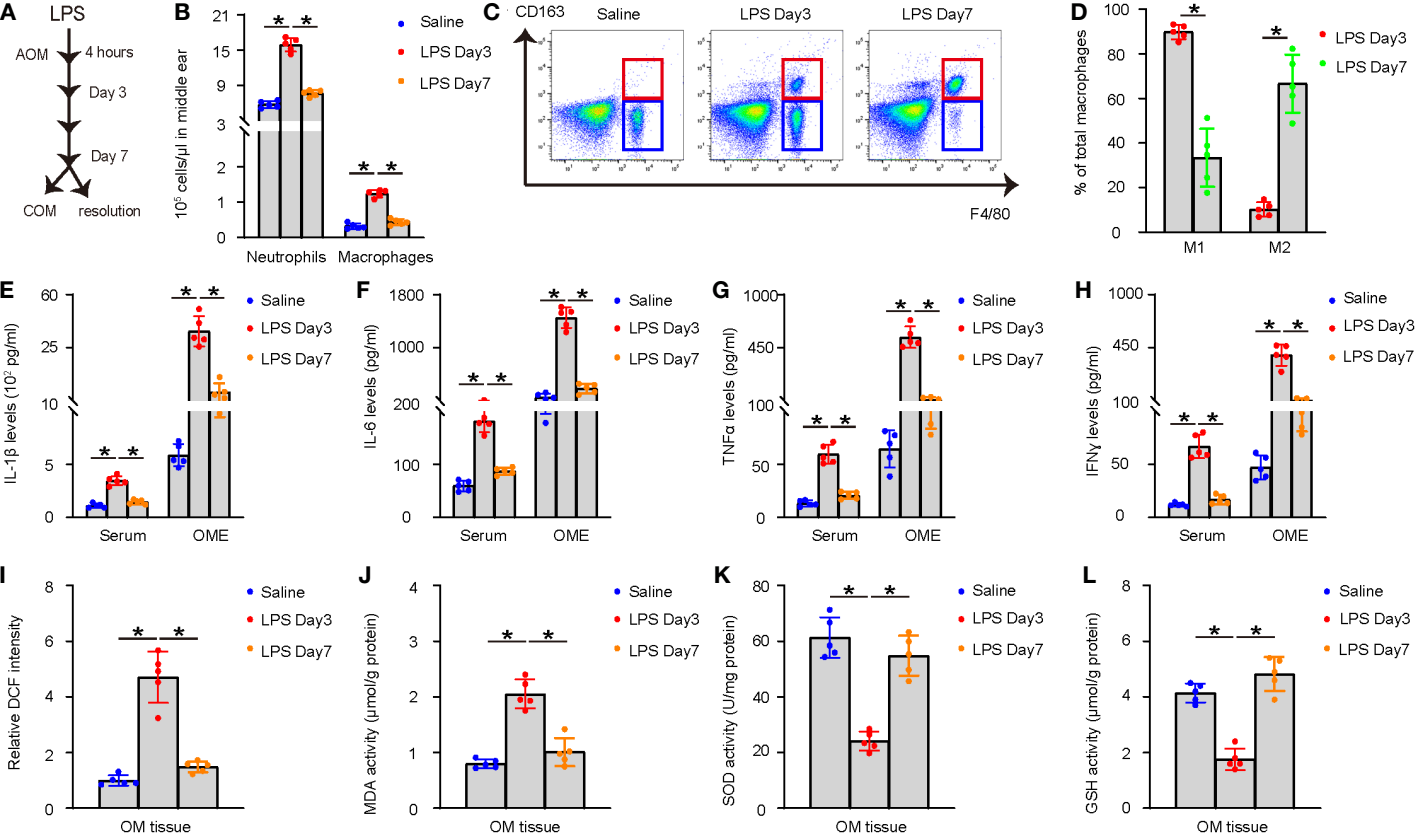
LPS injection into middle ear induces AOM in mice. **(A)** Schematic of the AOM model. A single high dose LPS (1.0 mg/ml) was given into the middle ear of the right ear of the mice. In this model, AOM is induced as early as 4 hours after LPS and lasts about 7 days before resolution of inflammation or progression into COM in a few mice. **(B–D)** OME was collected using myringotomy at day 3 and day 7 after LPS administration. **(B)** Neutrophils were manually counted. **(C)** F4/80+ macrophages were counted by flow cytometry. **(D)** Representative flow charts for analysis of macrophages (F4/80) and their polarization (CD163) by flow cytometry. **(E–H)** ELISA for IL-1β **(E)**, IL-6 **(F)**, TNFα **(G)**, and IFNɣ **(H)** in mouse serum and OME on saline-treated, and day 3 and day 7 LPS-treated mice. **(I–L)** The levels of ROS **(I)**, MDA **(J)**, SOD **(K)** and GSH **(L)** in the middle ear mucosae tissue. *p<0.05.

A well-established model for AOM was applied, for which a single high dose LPS (1.0 mg/ml) was given into the middle ear of the right ear of the mice. In this model, AOM is induced as early as 4 hours after LPS and lasts about 7 days before resolution of inflammation or progression into COM in a few mice (**Figure 1A**). The model was validated by examining the acute inflammatory response in the middle ear on day 3 and day 7 after LPS administration. OME was collected using myringotomy and the recruitment of inflammatory cells was analyzed. The results showed a significant increase in neutrophils and macrophages on day 3, followed by a significant reduction on day 7, as analyzed by manual counting and by flow cytometry, respectively (**Figure 1B**). Furthermore, most of the OME-macrophages on day 3 were CD163-negative M1 macrophages, while most of the OME-macrophages on day 7 were CD163-positive M2 macrophages (**Figures 1C, D**). In addition, the levels of major pro-inflammatory cytokines such as IL-1β, IL-6, TNFα, and IFNɣ significantly increased in mouse serum and OME on day 3 in LPS-treated mice, but these values significantly reduced on day 7 (**Figures 1E–H**). The levels of ROS, MDA, GSH, and SOD were also measured in the middle ear mucosae tissue to assess oxidation, showing a significant increase in ROS and MDA, and significant decreases in SOD and GSH on day 3. However, all these values returned to normal on day 7 (**Figures 1I–L**). These data demonstrate that LPS injection into the middle ear induces AOM in mice, which is resolved in most of the LPS-treated mice on day 7, although some variations in the examined values imply some cases of unsuccessful resolution of AOM.

### Reduced Nrf2 in macrophages leads to uncured AOM or development of COM

**Figure 2:**
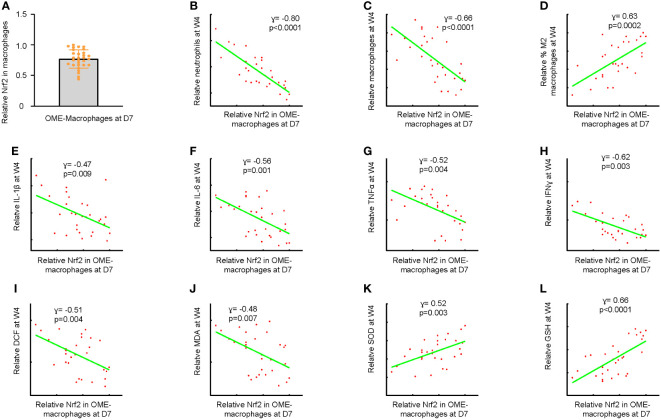
Reduced Nrf2 in macrophages leads to uncured AOM or development of COM. **(A)** Macrophages were sorted from OME at day 7 after LPS treatment in 30 mice by flow cytometry. ELISA for Nrf2 in the OME-macrophages were examined, shown as individual values. The maximum expression level of Nrf2 in macrophages was set as the reference point (=1). The expression levels of other samples were normalized to this reference value, allowing for a comparison of relative expression levels across samples. **(B–L)** The mice were kept for an additional 3 weeks (4 weeks after LPS) and examined for the recruitment of inflammatory cells and macrophage polarization in OME, the levels of major pro-inflammatory cytokines IL-1β, IL-6, TNFα and IFNɣ in mouse OME, as well as the levels of ROS, MDA, GSH and SOD in the middle ear mucosae tissue. Correlations of OME-macrophages at day 7 with neutrophil number in OME at 4 weeks after LPS **(B)**, macrophage number in OME at 4 weeks after LPS **(C)**, percentage of M2 macrophages in OME at 4 weeks after LPS **(D)**, OME-IL-1β levels at 4 weeks after LPS **(E)**, OME-IL-6 levels at 4 weeks after LPS **(F)**, OME-TNFα levels at 4 weeks after LPS **(G)** and OME-IFNɣ levels at 4 weeks after LPS **(H)**, and levels of ROS **(I)**, MDA **(J)**, SOD **(K)** and GSH **(L)** in the middle ear mucosae tissue at 4 weeks after LPS.

After confirming the induction of AOM by injecting a high dose of LPS, we treated 30 mice with LPS and extracted OME at day 7 through myringotomy. Macrophages were then sorted from OME by flow cytometry. The levels of Nrf2 in the OME-macrophages were examined using ELISA (**Figure 2A**). The mice were kept for an additional 3 weeks (4 weeks after LPS) and examined for the recruitment of inflammatory cells and macrophage polarization in OME, the levels of major pro-inflammatory cytokines IL-1β, IL-6, TNFα and IFNɣ in mouse OME, as well as the levels of ROS, MDA, GSH and SOD in the middle ear mucosae tissue. The correlation between the Nrf2 levels in OME-macrophages at day 7 and these parameters associated with AOM resolution was examined. Our data showed that lower Nrf2 levels in OME-macrophages at day 7 were significantly associated with more neutrophils in OME (p<0.0001, **Figure 2B**), more macrophages in OME (p<0.0001, **Figure 2C**), less percentage of M2 polarization of macrophages (p=0.0002, **Figure 2D**), higher levels of IL-1β (p=0.009, **Figure 2E**), IL-6 (p=0.001, **Figure 2F**), TNFα (p=0.004, **Figure 2G**) and IFNɣ (p=0.003, **Figure 2H**) in OME, higher levels of ROS (p=0.004, **Figure 2I**) and MDA (p=0.007, **Figure 2J**), and low levels of SOD (p=0.003, **Figure 2K**) and GSH (p<0.0001, **Figure 2L**) in the middle ear mucosae tissue at 4 weeks after LPS, indicating that reduced Nrf2 in OME-macrophages may lead to uncured AOM or the development of COM.

### Generation of macrophage-specific Nrf2-expressing vectors

**Figure 3:**
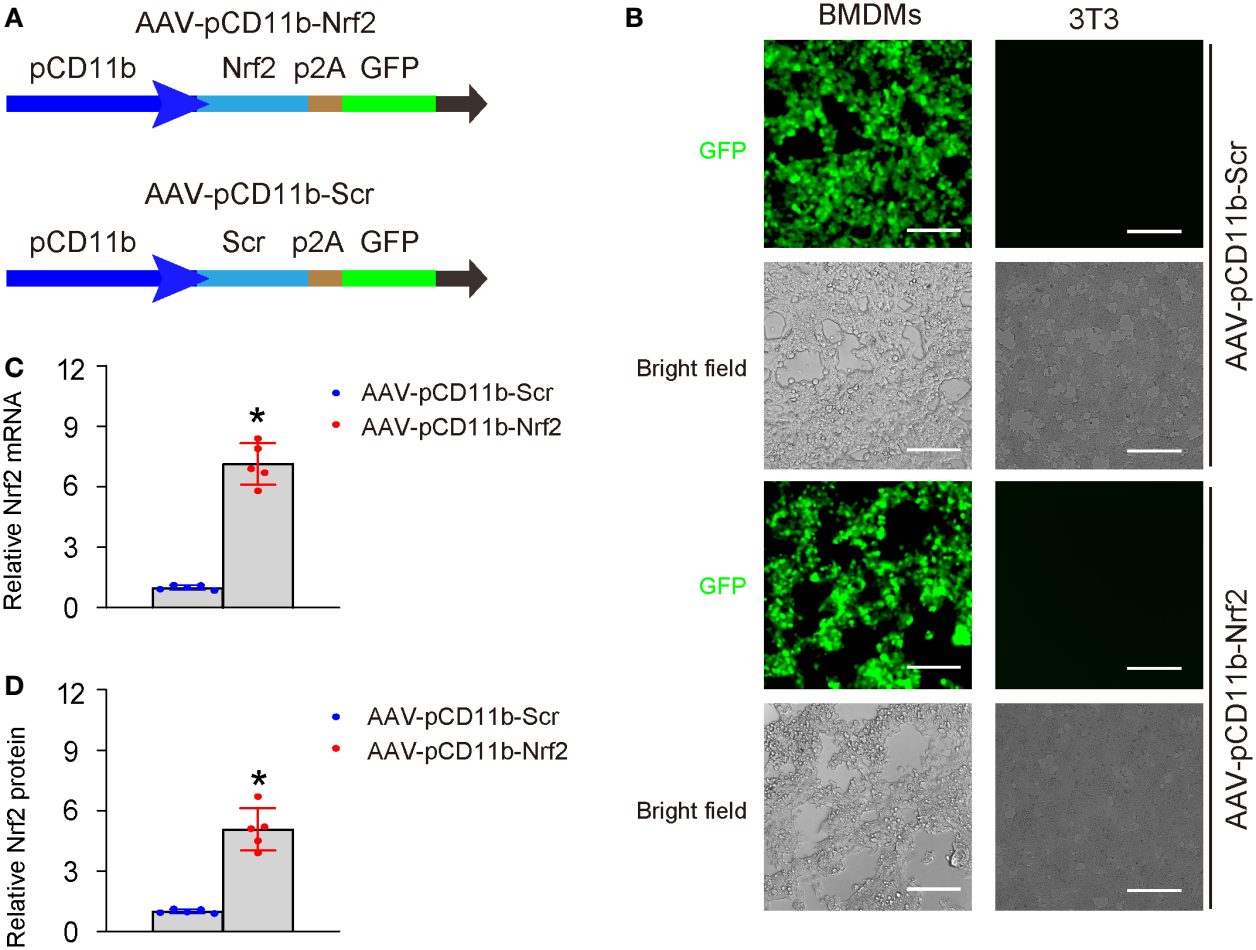
Generation of macrophage-specific Nrf2-expressing vectors. **(A)** Schematic of an AAV (serotype 6) containing Nrf2 under the macrophage-specific CD11b promoter (AAV-pCD11b-Nrf2) and a control AAV containing a scramble sequence (Scr) under the CD11b promoter (AAV-pCD11b-Scr). Both viruses also carried a green fluorescent protein (GFP) as a reporter linked with the transgene through a p2A construct, allowing for expression of both transgenes controlled by the CD11b promoter. **(B)** Transduced BMDMs and 3T3 cells *in vitro* shown by fluorescent and bright field channels. BMDMs, but not the 3T3 fibroblast cell line, displayed green fluorescence when transduced with both viruses, thus confirming the specificity of the CD11b promoter. **(C, D)** Quantification of levels of Nrf2 in transduced BMDMs, by RT-qPCR **(C)** and ELISA **(D)**. In panel D, the relative Nrf2 levels in BMDMs transduced with AAV-pCD11b-Scr were used as the reference point (=1), and the relative Nrf2 levels in BMDMs transduced with AAV-pCD11b-Nrf2 were calculated in comparison to this reference. *p<0.05. Scale bars were 70µm.

To investigate the effects of Nrf2 on macrophages and its role in AOM solution, we generated an adeno-associated virus (serotype 6) containing Nrf2 under the macrophage-specific CD11b promoter (AAV-pCD11b-Nrf2) and a control AAV containing a scramble sequence (Scr) under the CD11b promoter (AAV-pCD11b-Scr). Both viruses also carried a GFP reporter linked with the transgene through a p2A construct (**Figure 3A**). We successfully transduced BMDMs but not a fibroblast cell line 3T3 with both viruses (**Figure 3B**), validating the specificity of the CD11b promoter. Transduction with AAV-pCD11b-Nrf2 significantly increased the levels of Nrf2 in BMDMs, as determined by both RT-qPCR (**Figure 3C**) and ELISA (**Figure 3D**).

### Nrf2 induces M2-like polarization of macrophages

**Figure 4:**
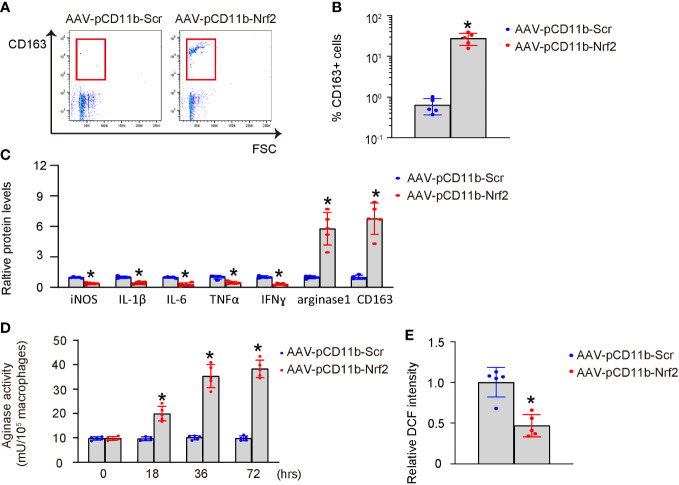
Nrf2 induces M2-like polarization of macrophages. BMDMs were transduced with AAV-pCD11b-Nrf2 or control AAV-pCD11b-Scr. **(A, B)** Flow cytometry for CD163 expression of transduced BMDMs, shown by representative flow charts **(A)** and by quantification **(B)**. **(C)** ELISA for iNOS, IL-1β, IL-6, TNFα, IFNɣ, arginase 1 and CD163 in transduced BMDMs. **(D)** Arginase activity. **(E)** ROS production. *p<0.05.

Next, we examined the effects of Nrf2 expression in BMDMs on macrophage polarization. AAV-pCD11b-Nrf2 was transduced into BMDMs, resulting in a significant increase in CD163+ BMDMs as shown by representative flow charts (**Figure 4A**) and quantification (**Figure 4B**). The expression of iNOS, IL-1β, IL-6, TNFα, and IFNɣ was significantly decreased, while the expression of arginase 1 and CD163 was significantly increased in BMDMs transduced with AAV-pCD11b-Nrf2 (**Figure 4C**). Additionally, transduction with AAV-pCD11b-Nrf2 resulted in a significant increase in arginase activity (**Figure 4D**) and a significant decrease in ROS production (**Figure 4E**) in BMDMs. Together, these data suggest that Nrf2 induces M2-like polarization of macrophages.

### Nrf2-macrophages improve survival of LPS-treated HMMECs

**Figure 5:**
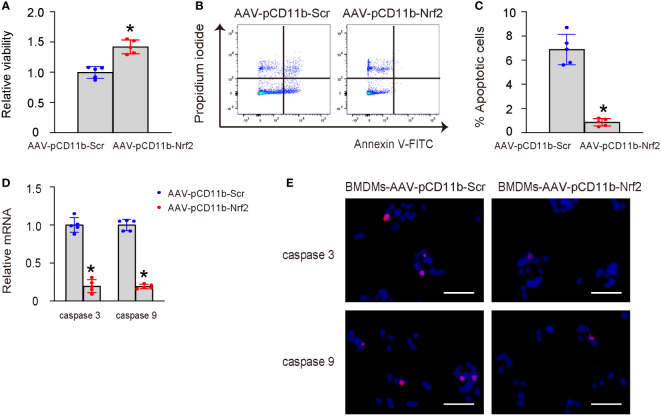
Nrf2-macrophages improve survival of LPS-treated HMMECs. HMMECs were treated with LPS, and then co-cultured with AAV-pCD11b-Nrf2-transduced or control AAV-pCD11b-Scr-transduced BMDMs. **(A)** CCK-8 assay for LPS-treated HMMECs co-culture with AAV-pCD11b-Nrf2-transduced or AAV-pCD11b-Scr-transuced BMDMs. **(B, C)** Annexin V assay for HMMECs, shown by representative flow charts **(B)** and by quantification **(C)**. **(D)** RT-qPCR for cleaved caspase 3 and caspase 9 in BMDMs. **(E)** Immunocytochemistry for cleaved caspase 3 and caspase 9 in BMDMs. *p<0.05. Scale bars were 50µm.

Human middle ear epithelial cells (HMMECs) are the primary cells that line the middle ear and play a vital role in the normal function of the ear. HMMECs were treated with LPS, and then co-cultured with AAV-pCD11b-Nrf2-transduced or control AAV-pCD11b-Scr-transduced BMDMs. Our data showed that co-culture with AAV-pCD11b-Nrf2-transduced BMDMs significantly increased the survival of LPS-treated HMMECs (**Figure 5A**), likely due to a reduction in apoptosis. This was shown by representative flow charts for an Annexin V apoptosis assay (**Figure 5B**) and by quantification (**Figure 5C**), as well as by mRNA levels and immunocytochemistry for apoptosis-associated proteins cleaved caspase 3 and caspase 9 (**Figures 5D, E**). Therefore, Nrf2-expressing macrophages improved the survival of LPS-treated HMMECs.

### Correction of Nrf2 expression in macrophages prevents transition from AOM to COM

**Figure 6:**
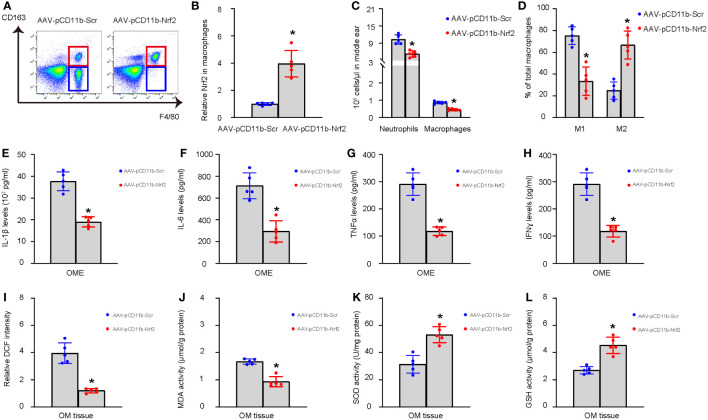
Correction of Nrf2 expression in macrophages prevents transition from AOM to COM. Immediate myringotomy-assisted injection of AAV-pCD11b-Nrf2 or control AAV-pCD11b-Scr into the middle ear cavity of selective LPS-treated mice with low Nrf2 levels in OME-macrophages at day 7 after LPS administration was performed. Afterwards, the mice were maintained for another 3 weeks (4 weeks after LPS) and examined again for the recruitment of inflammatory cells and macrophage polarization in OME, the levels of major pro-inflammatory cytokines IL-1β, IL-6, TNFα, and IFNɣ in OME, and the levels of ROS, MDA, GSH, and SOD in the middle ear mucosae tissue. Analysis was done at 4 weeks after LPS or 3 weeks after viral intervention. OME was collected using myringotomy. **(A)** F4/80+ macrophages were analyzed by flow cytometry, shown by representative flow charts. **(B)** ELISA for Nrf2 in OME-macrophages. The relative Nrf2 levels in OME-macrophages from mice transduced with AAV-pCD11b-Scr were used as the reference point (=1), and the relative Nrf2 levels in OME-macrophages from mice transduced with AAV-pCD11b-Nrf2 were calculated in comparison to this reference. **(C)** Number of neutrophils and macrophages in OME. **(D)** Quantification of macrophage polarization (CD163) by flow cytometry. **(E–H)** ELISA for IL-1β **(E)**, IL-6 **(F)**, TNFα **(G)**, and IFNɣ **(H)** in OME. **(I–L)** The levels of ROS **(I)**, MDA **(J)**, SOD **(K)** and GSH **(L)** in the middle ear mucosae tissue. *p<0.05.

Finally, we performed immediate myringotomy-assisted injection of AAV-pCD11b-Nrf2 or control AAV-pCD11b-Scr into the middle ear cavity of selective LPS-treated mice with low Nrf2 levels in OME-macrophages at day 7 after LPS administration. The group setup was randomized, and the relatively high or low values of Nrf2 were evenly distributed between the two groups. Afterwards, the mice were maintained for another 3 weeks (4 weeks after LPS) and examined again for the inflammatory cell recruitment and macrophage polarization in OME, the levels of major pro-inflammatory cytokines IL-1β, IL-6, TNFα, and IFNɣ in OME, and the levels of ROS, MDA, GSH, and SOD in the middle ear mucosae tissue. At 4 weeks, OME-macrophages were extracted for analysis to confirm the increase in Nrf2 levels by AAV-pCD11b-Nrf2 (**Figures 6A, B**). We found that re-expression of Nrf2 in OME-macrophages at day 7 significantly reduced inflammatory cells in OME (**Figures 6A, C**), increased M2 polarization of macrophages (**Figures 6A, D**), reduced the levels of IL-1β, IL-6, TNFα, and IFNɣ in OME (**Figures 6E–H**), reduced the levels of ROS and MDA (**Figures 6I, J**), and increased the levels of SOD and GSH (**Figures 6K, L**) in the middle ear mucosae tissue at 4 weeks. These findings suggest that correcting Nrf2 expression in macrophages prevents uncured AOM or development of AOM into COM.

## Discussion

The aim of the current study was to investigate the role of the Nrf2 signaling pathway in macrophage polarization and its effect on inflammatory resolution in AOM. We used an AOM model by injecting LPS into the middle ear cavity of the mice to determine the correlation between Nrf2 levels in OME-macrophages at the inflammatory resolution time point and the final inflammatory resolution. Our findings revealed that lower levels of Nrf2 in OME-macrophages at the inflammatory resolution time point were associated with poor inflammatory resolution and progression of AOM into COM. Indeed, the impaired resolution of inflammation was evidenced by the sustained presence of a high number of inflammatory neutrophils and macrophages, a continuous predominance of pro-inflammatory M1 macrophages, low transition to anti-inflammatory M2 macrophages, consistently elevated levels of pro-inflammatory cytokines such as IL-1β, IL-6, TNFα, and IFNɣ, as well as persistently high levels of tissue oxidation. Notably, the abnormal expression of Nrf2 was observed in patients with otitis media, suggesting a potential role of Nrf2 in the transition of AOM to COM (10). Our study further suggested that abnormal expression of Nrf2 may be attributable to macrophages, and play a significant role in the transition of AOM to COM.

Our study found that Nrf2 induced M2-like polarization of macrophages, which could be conducted through several signaling pathways. First, Nrf2 activates antioxidants by binding to ARE and subsequent activation of downstream target genes that promote M2 macrophage polarization (13). Second, Nrf2 interacts with toll-like receptor 4 (TLR4) signaling during inflammation (14). TLR4 is a TLR family member critical for innate immune response to infection (15). The TLR4 signaling pathway begins with the recognition of its ligand, which leads to the recruitment of adaptor proteins, such as MyD88 and TRIF, to the TLR4 complex (15). These adaptors, in turn, activate downstream kinases, such as IRAK and TBK1, which phosphorylate and activate transcription factors, such as NF-κB and IRF3, respectively (15). Activation of NF-κB leads to the expression of a wide range of pro-inflammatory cytokines, including TNFα, IL-1β, and IL-6, as well as chemokines, adhesion molecules, and other immune effector molecules (16). These factors promote the recruitment of immune cells such as neutrophils and macrophages to the site of infection or injury, where they undergo phenotypic changes such as M1 macrophage polarization for clearance of the pathogen (16). TLR4 and Nrf2 signaling pathways are interconnected and influence each other’s activity (17). Nrf2 inhibits NF-κB signaling by promoting the degradation of p65, inhibiting the phosphorylation and subsequent degradation of IκB, and competing with NF-κB for binding to CBP (18). In addition, Nrf2 also inhibits TLR4 signaling by blocking the production of pro-inflammatory cytokines and chemokines, contributing to the polarization of macrophages to M2 (18). Nrf2 can activate the PI3K/Akt pathway, which enhances the expression of anti-inflammatory cytokines and growth factors to promote M2 macrophage polarization (19).

Our study also found that Nrf2-mediated M2-polarization of macrophages improved the survival of co-cultured HMMECs treated with LPS *in vitro*. This improved survival resulted from the decrease in apoptosis of the LPS-injured HMMECs. M2 macrophages have been shown for their production and secretion of a variety of growth factors and cytokines that improve antiapoptotic cell survival in the microenvironment, including basic fibroblast growth factor (bFGF), IL-10, transforming growth factor-beta (TGF-β), IL-13, IL-4, EGF, platelet-derived growth factor (PDGF), hepatocyte growth factor (HGF), vascular endothelial growth factor (VEGF), insulin-like growth factor 1 (IGF-1) and macrophage-colony stimulating factor (M-CSF) (20). Hence, M2 macrophages may improve antiapoptotic cell survival in the microenvironment through these growth factors and cytokines (21).

In conclusion, our study demonstrates that restoration of Nrf2 in OME-derived low-Nrf2-expressing macrophages through a macrophage-specific gene targeting method can significantly inhibit the transition of AOM to COM in experimental mice. This approach could be a promising translational strategy to prevent the development of COM in AOM-patients.

## Data availability statement

The original contributions presented in the study are included in the article/supplementary material. Further inquiries can be directed to the corresponding author.

## Ethics statement

The animal study was reviewed and approved by Shanghai Children’s Hospital.

## Author contributions

WF, HX, CS, JF, and XL did data acquisition and analysis; WF and XL performed study conception and applied for supportive funding. WF wrote the manuscript. All authors reviewed the manuscript and agreed with the publication. XL is the guarantee of this study. All authors contributed to the article and approved the submitted version.
